# Dr. J. Thad Johnson

**Published:** 1883-08

**Authors:** 


### DR. J. THAD JOHNSON.

We have received a card from this excellent physician, by which we are glad to learn that his health is so far restored that he will be able, next fall, to resume his usual professional duties. Richard is himself again.

